# The roles of family and friends in the immobility decisions of university graduates staying in a peripheral urban area in the Netherlands

**DOI:** 10.1002/psp.2392

**Published:** 2020-11-02

**Authors:** Jonne A. K. Thomassen

**Affiliations:** ^1^ Population Research Centre, Faculty of Spatial Sciences University of Groningen Groningen The Netherlands

**Keywords:** family, friends, immobility, peripheral urban area, staying, university graduates

## Abstract

Highly educated individuals constitute great assets for regional development and economic growth. Nevertheless, young university graduates are relatively geographically mobile and less likely to stay in peripheral regions. Based on semi‐structured, life‐calendar interviews, this study explored the immobility decisions of graduates who have stayed in a peripheral urban area in the Netherlands where they completed their university education. The study specifically focused on the roles of family and friends in the staying processes of these young adults. The results indicate that the decision to stay was frequently and consciously re‐evaluated by some, whereas for others, it resulted from a ‘lack of triggers’ for moving elsewhere. Notably, the interviews revealed that family and friends act as more than motives for staying or deterrents to migration. On various occasions, family and friends had played crucial roles as advisors, influencers, triggers, exemplars and facilitators in the staying processes of highly educated young adults.

## 1 INTRODUCTION

Highly educated individuals embody strong human capital. For this reason, they are considered great assets in regional development and endogenous economic growth (Faggian, 2006; Faggian & McCann, 2009; Lucas, 1988; Romer, 1986). However, university graduates tend to move frequently (Bernard, Bell, & Charles‐Edwards, 2014; Chudnovskaya & Kolk, 2017; Lundholm, 2007; Rogers & Castro, 1981), over long distances (Champion & Shuttleworth, 2017; van Ham, 2002) and towards central, economic ‘escalator regions’ (Fielding, 1992; Findlay, Mason, Houston, McCollum, & Harrison, 2009). For the graduates, these centre‐oriented moves increase the chances of finding a good match between regional labour markets and their recently obtained degrees (Büchel & van Ham, 2003; Hensen, De Vries, & Cörvers, 2009; Iammarino & Marinelli, 2015; Venhorst & Cörvers, 2018), but for peripheral regions, these moves may present a loss of human capital (for the Netherlands, see Latten, Kooiman, & Bontje, 2017; Venhorst, 2013; Venhorst, van Dijk, & van Wissen, 2010, 2011).

Focusing on this strikingly mobile profile, few studies have investigated the staying processes of highly educated young adults in peripheral regions (for the Netherlands, see Stockdale, Theunissen, & Haartsen, 2018; Venhorst, 2013). Even more so, the emerging immobility literature has yet to come to a consensus over the factors influencing immobility decisions. So far, living close to friends (Belot & Ermisch, 2009) and family (Clark, Duque‐Calvache, & Palomares‐Linares, 2017; Ermisch & Mulder, 2018; Michielin, Mulder, & Zorlu, 2008; Mulder & Malmberg, 2011, 2014; Zorlu, 2009) have been put forward as primary factors in decreasing the propensity to migrate. This study responds to a call for more thorough investigations into the dynamics between staying behaviour and social networks (Hjälm, 2014) required for strengthening the theorisation on the roles of family and friends in residential decisions (Faist, 1997; Mulder, 2018). It does so by investigating the roles of family and friends in the staying processes of highly educated young adults in a peripheral urban area in the Netherlands. Notably, the study treats spatial immobility as an active process in one's residential trajectory, signalling a newly emerging perspective within the migration literature (e.g., Coulter, van Ham, & Findlay, 2016; Haartsen & Stockdale, 2017; Hjälm, 2014; Stockdale et al., 2018).

Data for this study stem from semi‐structured, life‐calendar interviews with 15 Maastricht University alumni who have stayed in Maastricht since completing their tertiary education trajectories. The city of Maastricht is located in the province of Limburg, which is a peripheral area in the south of the Netherlands. The city and the province typically experience out‐migration of highly educated young adults (e.g., Hooijen, Meng, Reinold, & Siegel, 2017; Venhorst et al., 2010). The interviews revealed a diverse range of factors that may trigger re‐evaluations of the decision to stay as well as the role of a ‘lack of triggers’ for continued staying behaviour. More specifically, the interviews confirm that living close to family and friends can be an important motive for staying, as well as a deterrent to migration. The most significant contribution to the literature may be found in the descriptions of family and friends serving as advisors, influencers, triggers, exemplars and facilitators in the staying processes of highly educated young adults.

## 2 THEORY

### 2.1 The immobility literature

A small but growing body of literature has started to advance our understanding of nonmigration experiences and processes of spatial immobility. First and foremost, the immobility literature has pondered the question as to whether spatial immobility or spatial mobility is the normative—also referred to as sedentarism and nomadism respectively (e.g., Cresswell, 2006; Di Masso et al., 2019; Hjälm, 2014; Jónsson, 2011; Sheller & Urry, 2006). Furthermore, numerous authors have called for more empirical studies on immobility experiences in their own right, seeing this as an underrepresented topic within the broader migration literature (e.g., Cooke, 2011; Coulter, van Ham & Findlay, 2013, 2016; Hanson, 2005; Kothari, 2003; Mulder, 2018; Schewel, 2019; Stockdale & Haartsen, 2018). Subsequently, the immobility literature has been expanding with qualitative, quantitative and mixed‐method explorations of the motives for spatial immobility and various other aspects of the staying process (e.g., P. A. Fischer, Holm, Malmberg, & Straubhaar, 2000; P. A. Fischer & Malmberg, 2001; Morse & Mudgett, 2018; Ní Laoire, 2005; Preece, 2018; Reeves, 2011; Schewel, 2015; Winters, 2012).

These empirical endeavours have given rise to conceptualisations of different types of spatial immobility. For instance, spatial immobility has been defined according to various time dimensions, ranging from one‐day immobility (Adeel & Yeh, 2018) to long‐term or lifelong immobility (Erickson, Sanders, & Cope, 2018; Hjälm, 2014). Immobility has simultaneously been explored in various spatial contexts using different place‐dimensions, such as residential or neighbourhood immobility (Burholt & Sardani, 2018; Clark et al., 2017; Hjälm, 2014), regional or rural–urban immobility (Erickson et al., 2018; Haartsen & Stockdale, 2017; Stockdale et al., 2018) and international immobility (Alberts & Hazen, 2005; Baykara‐Krumme & Platt, 2018; Bobek, 2020). Furthermore, focusing on the agency of stayers has revealed not only voluntary and satisfied staying behaviour but also involuntary and reluctant staying behaviour (e.g., Ferro, 2006; Haartsen & Stockdale, 2017; Jónsson, 2008; Lubkemann, 2008; Mata‐Codesal, 2018; Schewel, 2019; Ye, 2018). Nevertheless, the mechanisms behind the decision to stay and the roles of others in the decision‐making process remain less understood (Clark & Lisowski, 2019).

### 2.2 The decision to stay

One of the first qualitative studies on lifelong staying behaviour put forward the idea that ‘staying is by no means just something that “happened to” the informants’ (Hjälm, 2014, p. 577). In fact, that study revealed that the decision to stay is commonly ‘an active and informed choice’ and may be re‐evaluated and renegotiated over the life course (Hjälm, 2014, p. 577). Since then, other studies have confirmed that immobility decisions are not one‐off events but consciously revisited (e.g., Haartsen & Stockdale, 2017; Stockdale et al., 2018). For example, some informants in a study by Haartsen and Stockdale (2017) were ‘convinced stayers’, yet they anticipated re‐evaluating their staying decision at the onset of future life‐course changes or life events, such as transitioning to an empty‐nest life phase or reaching old age and becoming less physically abled. These findings provide evidence for an idea from the migration literature that residential processes are fluid and never complete (Halfacree, 2004, 2018), but they are novel in treating nonmigration as an active component in these processes. The findings further suggest that nonmigration experiences should be positioned more fully within one's biography to take into account the role of the life course and key events, as has been argued for migration experiences (Barcus & Halfacree, 2018; Halfacree & Boyle, 1993).

By positioning staying processes more fully within one's biography, the existing immobility literature has framed past, present and future life events as potential triggers for a re‐evaluation of the decision to stay (e.g., Haartsen & Stockdale, 2017; Stockdale et al., 2018; Ye, 2018). Haartsen and Stockdale (2017) have further suggested that a history of migration may trigger more conscious re‐evaluations of one's residential situation because movers are already familiar with the re‐evaluation process. To this end, it should be noted that each re‐evaluation, now and in the future, may result in either staying or moving behaviour. Hence, Stockdale et al. (2018, p. 6) have argued that conscious renegotiations of the decision to stay signal that staying processes are ‘in a state of flux’.

### 2.3 The roles of family and friends in the decision to stay

At the same time, Hjälm (2014, p. 579) has argued that the decision to stay ‘does not occur in isolation but is connected to other life projects and people’. In order to understand how immobility decisions or re‐evaluations thereof are influenced by others, the existing framework regarding the decision to stay should include the notion of ‘linked lives’. The ‘linked lives’ approach encourages abandoning the individual as the sole unit of analysis and, instead, views life events as experienced together with others, such as family and friends (Elder, 1994; Elder, Johnson, & Crosnoe, 2003). Life events that are typically experienced with others during young adulthood include leaving the parental home, starting to cohabit, getting married, having children, leaving a relationship, experiencing loss and parental divorce.

Some of these collective experiences may well affect the relationship between young adults and their parents, siblings, partners or housemates (e.g., Bidart & Lavenu, 2005). Notably, leaving the parental home may decrease the frequency of face‐to‐face contact and change the type of support that is given and received between young adults and their parents. It could be argued that such changes to the parent–child relationship may be more prominent among young adult migrants than those who stay in their hometown. Nevertheless, Hjälm (2011, 2014) notes that a decision to stay may affect the social network too, because staying may lead to extensive longstanding social networks as well as feelings of isolation if family and friends move away. These contrasting immobility experiences led Hjälm (2011, 2014) to call on future research to explore the dynamics between staying behaviour and the social network in more detail.

The ‘linked lives’ approach further suggests that family and friends may influence the course of one's life events (Elder, 1994; Elder et al., 2003) and this raises the question of how linked lives affect one's residential trajectory. Thus far, the literature on family ties in relation to mobility behaviour has found that living close to family increases the likelihood of staying and of returning, as well as decreasing the propensity to migrate (Clark et al., 2017; Ermisch & Mulder, 2018; Michielin et al., 2008; Mulder & Malmberg, 2011, 2014; Niedomysl & Amcoff, 2011; von Reichert, Cromartie, & Arthun, 2014; Zorlu, 2009). Similarly, living close to friends decreases the likelihood of migrating (Belot & Ermisch, 2009). Although, on a note of caution, friend‐related motives are rarely mentioned separately and are mostly found in combination with family‐related motives (Gillespie & Mulder, 2020). These findings have been attributed to the fact that family and friends constitute a large part of people's social capital (Bengtson, 2001; Conkova, Fokkema, & Dykstra, 2018; Rossi & Rossi, 1990) and that geographic proximity is crucial for maintaining strong relationships with family and friends because these require opportunities for face‐to‐face contact and supportive exchange (Bordone, 2009; Greenwell & Bengtson, 1997; Hank, 2007; Hjälm, 2012; Knijn & Liefbroer, 2006; Meil, 2006; Michielin et al., 2008; Mulder & van der Meer, 2009). Thus, living close to family and friends is expected to constitute a motive for staying and act as a deterrent to migrating in the immobility decisions of young, highly educated adults.

Another way in which family and friends have been found to exert influence on one's residential behaviour is through socialisation. Socialisation theories suggest that one's preferred behaviour is informed by choices, preferences, attitudes and examples set by others. Residential experiences during childhood, experienced together with household members, are found to be positively related to similar residential behaviour in adulthood (Ærø, 2006; Bernard & Vidal, 2020; Blaauboer, 2011). On the family level, the transnational migration literature has shown that migration behaviour is passed down through generations in a family (e.g., Guveli et al., 2016). Similar findings have been presented regarding passed‐down migration intentions of young adults in peripheral regions (Thissen, Fortuijn, Strijker, & Haartsen, 2010). On a more aggregated scale, migration has been found to trigger further migration, known as the ‘cumulative causation of migration’ (Massey, 1990). The immobility decisions of the highly educated young adults are thus expected to be informed by the residential behaviour of their social environment, including family and friends.

### 2.4 Other motives for staying

Of course, staying behaviour may be motivated by factors other than family and friends. Some studies have highlighted the role of personal factors, the physical environment and the cultural environment as motives for staying (e.g., Alberts & Hazen, 2005; Haartsen & Stockdale, 2017). Most notably, numerous empirical studies have found that the length of stay increases the likelihood of continuing to stay (Clark et al., 2017; P. A. Fischer et al., 2000; P. A. Fischer & Malmberg, 2001; Thomas, Stillwell, & Gould, 2016). This has been attributed to the idea that one feels more resistance towards moving as the duration of stay increases, also known as ‘cumulative inertia’ (e.g., Huff & Clark, 1978). This is also why continued staying behaviour has been associated with increased feelings of place attachment (Clark et al., 2017; Di Masso et al., 2019), rootedness (Cooke, 2011; C. S. Fischer, 2002) and the accumulation of local ties and location‐specific capital (Blaauboer, 2011; David, Janiak, & Wasmer, 2010; P. A. Fischer & Malmberg, 2001; Mulder & Malmberg, 2014). Others have identified staying behaviour as the result of a ‘lack of triggers’ or a lack of reasons for moving (Mulder, 2006), which may reflect the absence of push factors or ‘residential stress’ in the current location and of more attractive places elsewhere (Hjälm, 2014; Huff & Clark, 1978). Finally, applying a life course approach to long‐term immobility, Hjälm (2014) found that stayers mention multiple reasons or rationales for the decision to stay at various phases of the life course. Indeed, Haartsen and Stockdale (2017) have since then showed that there may be a temporal dimension to one's stated motives.

### 2.5 Context: Spatial immobility among university graduates in Maastricht

The motivation for this study's focus is based on the premise that spatial immobility is relatively uncommon among highly educated, young adults. In part, this was deduced from the universal age profile of migration, which has revealed that migration propensities are highest during young adulthood (Bernard et al., 2014; Rogers & Castro, 1981). In addition, more highly educated individuals show higher migration frequencies (Chudnovskaya & Kolk, 2017; Lundholm, 2007), tend to move over longer distances (Champion & Shuttleworth, 2017; van Ham, 2002) and towards centres of job opportunities, also known as escalator regions (Fielding, 1992; Findlay et al., 2009; Venhorst et al., 2010, 2011).

In the Netherlands, access to jobs tends to be worst in the peripheral regions (van Ham, Mulder, & Hooimeijer, 2001) towards the southern borders with Belgium and the eastern borders with Germany. Access to jobs and further opportunities for upward job mobility are highest in the Randstad (van Ham et al., 2001), the central urban region that includes some of the country's primary cities: Amsterdam, Rotterdam, The Hague and Utrecht. In Fielding's (1992) terminology, the Randstad can be considered the Netherlands' primary ‘escalator region’ (van Ham et al., 2001). Accordingly, a significant number of students and graduates from the Dutch peripheral regions move to the Randstad (Latten et al., 2017; Venhorst, 2013; Venhorst et al., 2010, 2011).

The spatial context for this study is Maastricht: a university city located in a peripheral area in the south of the Netherlands on the border with Belgium. It is the provincial capital and the largest urban conurbation in the Dutch province of Limburg. The city houses a young, internationally competitive university, which has frequently ranked in the top 10 of the Young University Rankings (Times Higher Education, 2020). Despite this, most of its graduates move towards the Randstad or abroad upon graduation (Hooijen et al., 2017; Venhorst et al., 2010). The university's alumni who decide to stay in Maastricht embody strong human capital that is crucial for Limburg's development and economic growth (e.g., Venhorst et al., 2010).

## 3 DATA AND METHODS

### 3.1 The recruitment process and sample

This study set out to explore the immobility decisions of Maastricht University alumni who have stayed in Maastricht since completing their tertiary education. The author requested members of an alumni community on social media to respond if they were interested in taking part in the study. The request contained a decision‐tree, which ensured that potential interviewees met the following five selection‐requirements: (1) to hold a master's degree, or equivalent,^1^ from Maastricht University; (2) to have lived in Maastricht while completing their master's degree; (3) to have stayed in Maastricht since graduating from Maastricht University; (4) to be no more than 40 years old; and (5) to be born in the Netherlands or the bordering Belgian province of Limburg. The author received over 30 responses of which 15 were from young adults who met all the requirements and were able to meet for an interview. Some self‐selection bias in the recruitment process may have been created by targeting members of an alumni‐group as these tend to foster particularly positive feelings towards their former university or its city. We targeted young adults who were born in the Netherlands or the bordering Belgian province of Limburg to ensure that all interviews could be held in one language, namely, Dutch. This reduces validity issues related to the interpretation and translation of language‐specific expressions (e.g., van Nes, Abma, Jonsson, & Deeg, 2010). Nevertheless, the staying processes of non‐Dutch‐speaking graduates in Maastricht are equally relevant and may be investigated in future studies.

Table 1 shows some key individual characteristics of the 15 interviewees. Eight females and seven males were interviewed, all between the ages of 24 and 40. Their names, in the first column, are pseudonyms to ensure confidentiality. The second column shows that eight interviewees were born in the Dutch province of Limburg (LIM), four in other Dutch provinces (NL) and three in the Belgian province of Limburg (BE). Furthermore, the fifth column shows that five interviewees had lived in the city of Maastricht at some point before the age of 18; two of these had always lived in Maastricht and three had moved away from Maastricht during childhood and returned during adulthood (not shown in table). The remaining 10 interviewees all moved to Maastricht at or after the age of 18. The final column shows the consecutive number of years that each interviewee had lived in Maastricht as adults, counting from the date of the interviews taking place in May, June and July of the year 2018. The interviewees' full staying periods may include additional years lived in Maastricht as children and varied from 3 years to a lifetime (not shown in table).

**TABLE 1:** Individual characteristics of the 15 interviewees

Name	Place of birth	Sex	Age	Lived in Maastricht before age 18	Graduated (year)	Consecutive years living in Maastricht since age 18
Thomas	Dutch province of Limburg (LIM)	Male	27	Yes	2017	9
Levi		Male	26		2013	8
Tom		Male	29		2016	7
Anouk		Female	28		2015	3
Hugo		Male	27		2017	3
Nina		Female	37	No	2006	12
Steffie		Female	33		2012	8
Julia		Female	29		2016	7
Rick	The Netherlands, other than Limburg (NL)	Male	40		2005	21
Lucas		Male	38		2004	21
Karin		Female	32		2009	14
Anne		Female	24		2016	3
Sandrine	Belgian province of Limburg (BE)	Female	35		2007	10
Sara		Female	28		2012	10
Serge		Male	28		2013	10

### 3.2 Semi‐structured, life‐calendar interviews

The author opted for combining traditional, qualitative semi‐structured interviews with life‐calendar grids. Qualitative interviewing methods help to reveal in‐depth information about interviewees, whereas calendar‐based interviewing methods have been developed to increase the richness of data in quantitative life‐course research (Belli, 1998; Belli, Shay, & Stafford, 2001; Freedman, Thornton, Camburn, Alwin, & Young‐DeMarco, 1988). More recently, life‐calendar grids have been used to provide direction and chronological structure to qualitative studies and to capture the timing and interlinkages of life events more accurately (e.g., Barbeiro & Spini, 2015, 2017; Kõu, van Wissen, van Dijk, & Bailey, 2015). These two methods were combined to allow a biographical approach to the staying processes of the interviewees, which was deemed crucial for treating nonmigration as an active component in their residential trajectories. In this respect, the life‐calendar grid was utilised as a tool for stimulating long‐term memory (Barbeiro & Spini, 2015, 2017; Bradburn, Sudman, & Wansink, 2004). This was expected to aid the recollection of past, present and future life events, which are known to trigger re‐evaluations of immobility decisions (e.g., Haartsen & Stockdale, 2017; Hjälm, 2014; Stockdale et al., 2018; Ye, 2018). Nevertheless, interviewers are cautioned about the many types of recall bias and recall errors that may arise in retrospective recollections (e.g., Auriat, 1991; Bell, 2005).

The author started out by designing a semi‐structured interview guide with open‐ended questions and probing examples regarding the interviewees' residential, education and career trajectories, followed by other important life events, the composition of their social network, and general impressions of the living environment. The interview guide continued by asking more specific questions pertaining to the staying process; for instance, about reflections on the staying period, motives for the decision to stay and residential intentions for the future. Following this, a life calendar was designed that corresponded to the subthemes in the interview guide (see Table 2 for an example of a blank life‐calendar grid). The author conducted three test interviews before finalising the interview guide and the outline of the life‐calendar grid; to enhance legibility and allow the interviewee to read and correct the interviewer's interpretations, it was found that the life‐calendar grid was to spread across two landscape‐oriented A3 sheets. At the start of each interview, the life‐calendar grid was personalised by entering the interviewee's age and corresponding calendar years in the first two rows. As such, each completed life‐calendar grid represented a personalised timeline that was annotated according to the interviewees' retrospective recollections of life events and experiences, thus containing extensive information about the interviewee's life course and staying period.

**TABLE 2:** Example of a blank life‐calendar grid

Year (birth to present)															
Age	0	1	2			18	19			25			38	39	40
Municipality of residence															
Urban or rural environment															
Homeowner or renter															
Household composition															
Type of education (i.e., primary, secondary and tertiary)															
Level of education															
Location of education institute															
Employed/unemployed															
Type of job															
Location of job															
Life events															
Life trajectories															
Changes to personal network															
Receiving or giving support or any other roles of network															
Other social contacts															
Cultural activities or cultural environment															
Physical environment															
Future plans															
Attitudes/expectations towards staying and moving															
Motives for moving or returning to Maastricht															
Motives for staying in Maastricht															
Miscellaneous notes															

*Note*: Several columns of the life‐calendar grid have been removed to fit the page margins. The original life‐calendar grids contained all person‐years between ages 0 and 40 and were spread across two landscape‐oriented A3 sheets to enhance legibility.

The interviewees were offered a choice of where to meet and eight chose to be interviewed in the author's temporary home in Maastricht and seven were interviewed at their workplace in the area of Maastricht. The interviews were recorded and lasted between 50 and 105 min. Here, one should note that the author studied at Maastricht University and lived in Maastricht several years prior to the interviews. This shared identity, residential location and pre‐existing knowledge of location‐specific aspects may have influenced the statements given by the interviewees. For example, shared knowledge can help the interviewer understand the interviewees' location‐specific references, but it can also negatively affect the richness of the recorded data if interviewees refrain from offering detailed information because they assume the information is already known to the interviewer.

### 3.3 Analytical approach

The interviews were transcribed verbatim and the life‐calendar grids were photographed and digitised. Except for ‘Maastricht’, all names, locations and other identifiers were changed to pseudonyms or general geographical areas to ensure privacy and anonymity. Following this, the digital transcripts and life‐calendar grids were coded and analysed in Atlas.ti software. The biographical approach included linking the narratives of the interviewees to specific moments and events in their life courses or staying periods; visualising and mapping out the decision‐making processes; and adding in‐depth information about the various factors that influenced mobility and immobility decisions. As recommended for non‐English qualitative data (see van Nes et al., 2010), relevant extracts were translated to English with the help of a professional language editor towards the end of the analytical process.

Three main themes arose from deductive and inductive coding (i.e., through a thematic analysis), namely, the decision to stay; the roles of the microcontext, mesocontext and macrocontext in the staying process; and the stated motives for staying. The last theme was the direct product of the answers to the question, ‘What is your main motive for staying in Maastricht?’, revealing a wide range of motives and considerations that could be reduced to four overarching factors: intrinsic values; career trajectories; family‐ and friend‐related considerations; and the physical and social environment. The current article reports on the findings related to one main theme and two subthemes as these are particularly relevant to the current debates on spatial immobility. The main theme described is the decision to stay, and the two subthemes are family‐ and friend‐related motives for the decision to stay and the role of family and friends in the staying process. Indeed, the data collected went beyond these three aspects, and our intention is to report findings related to the remaining themes and stated motives in the future to further advance our understanding of the staying processes of highly educated young adults.

## 4 RESULTS

### 4.1 What triggers a re‐evaluation of the decision to stay?

In line with the literature, some interviewees mentioned key life events as triggers for re‐evaluating their decision to stay. For example, for Hugo (LIM, 27), the start of his master's programme in Maastricht marked the moment when he decided, ‘I am going to stay here’. He described graduating from the master's programme just 1 year later as standing at another important ‘crossroads’ where he re‐evaluated his staying decision.

Further, the transcripts of some of the interviews revealed additional factors that had triggered a re‐evaluation of the decision to stay, but these could less readily be conceptualised as typical demographic life events. Rather, the additional triggers represented unique, personal events that the interviewee considered decisive in their re‐evaluation of the decision to stay. For instance, Rick (NL, 40) had re‐evaluated his decision to stay when he was presented with a job offer in the Randstad, and Steffie (LIM, 33) linked her decision to stay in Maastricht to travelling abroad and a collectively experienced life event of her partner:

There was a time when I deliberated about moving to [another job in the Randstad] and really change course in life. During that time, [my partner and I] seriously considered moving. […] We looked at some houses and had a real estate agent inspect our house. But when that same job […] also became available in South Limburg, I decided […] to apply here in South Limburg. (Rick, NL, 40)

In hindsight, and broadly speaking, two moments have been decisive for me since my master's degree. […] During [a trip abroad], I did some soul‐searching and asked myself: ‘what is most important to me’? […] And, [at a later moment], my partner decided to move to Maastricht. […] Those two moments have caused me to […] stay in Maastricht. (Steffie, LIM, 33)

For some, re‐evaluating the decision to stay had been a frequent, conscious exercise. For example, Lucas (NL, 38) had moved to Maastricht for his studies but, since graduating, has re‐evaluated his decision to stay in Maastricht on an annual basis. He did not mention any specific life events or personal events that had served as triggers for these frequent re‐evaluations. Rather, questioning whether his current location was still the right place for him seemed to be an exercise born out of habit or routine:

Basically, I have asked myself whether I am in the right place or if I have to move on a yearly basis. And, every time, I was able to answer that question with: ‘I am completely in the right place here’. Thus, I have made that decision each year anew. (Lucas, NL, 38)

Levi (LIM, 26), on the other hand, did not reveal any moments when he had consciously decided to stay in Maastricht. He had lived in Maastricht his entire life and explicitly mentioned that continuing to stay in Maastricht had been ‘very logical’ because ‘there had not been any triggers to leave Maastricht’. This idea that a ‘lack of triggers’ results in continued staying behaviour is also seen in the literature, where it is stated that external triggers are required to start considering migration (Mulder, 2006). Moreover, comparing the transcripts of Levi (LIM, 26) and Lucas (NL, 38) revealed the possible effects of having previous migration experiences on re‐evaluating the decision to stay. This relates to a finding by Haartsen and Stockdale (2017), who suggested that people with a history of migration may reflect on their staying behaviour more frequently or more consciously than natives and long‐term stayers because they are familiar with exercising such decisions and such re‐evaluations.

### 4.2 Open to moving, but likely to stay

The recurring re‐evaluations provide evidence for the idea that the decision to stay is not necessarily a one‐off event. The interviewees seemed to be aware that each evaluation of the decision to stay—now and in the future—may result in either moving or staying. The interviewees primarily demonstrated this awareness by mentioning circumstances that could potentially trigger a re‐evaluation or move in the future. For example, despite having experienced a ‘lack of triggers’ so far, Levi (LIM, 26) expressed himself open to moving in the future if an adequate trigger, such as a dream job, presented itself:

I have always been open to that. […] If I find a job [outside of this area] to which I react: ‘Yes, this is it!’ Yes, well, then I would be willing to move. See, I have everything here, but I do not think to myself: ‘I am tied to this place and I will never leave’. […] The only thing is, there needs to be a specific trigger for leaving …. (Levi, LIM, 26)

Even more so, some interviewees had actively created circumstances that would enable a future move. For example, after graduating, Lucas (NL, 38) and his partner lived in rental dwellings for about 5 years: ‘Also just for keeping our options open, because as long as we were renting, we would be able to leave at any moment’. Similarly, Tom (LIM, 29) said he was keeping his options open regarding job opportunities outside of the region:

I continue to look at other opportunities. I hear from friends that it can be very important to stay in one place, but it can create a sense of becoming stuck in the place or getting stuck in a rut. (Tom, LIM, 29)

Despite being open to moving in the future, the outcome of a re‐evaluation may become more skewed towards continued staying behaviour over time. This is hinted at in the above quote by Tom (LIM, 29) when he notes that, by staying in one place, you can get ‘stuck in the place’. The idea that people are more likely to stay as the duration of the current stay increases was reiterated in the statements of other interviewees. For example, Steffie (LIM, 33) stated that, over time, strengthened ties and roots make it more difficult to move in the future, and Lucas (NL, 38) stated that once you decide to stay for the long‐term, you start settling down more:

Yes, you see, you tend to strengthen ties to an area. Or, you grow more and more roots there as it were. Those things make it more difficult to consider leaving and uprooting your life again. (Steffie, LIM, 33)

And that's when we knew we were in the right place, and also when we bought the house. That's the moment we decided that we were going to stay here for the long‐term. Those things are interdependent and make you feel like settling down a little bit more. (Lucas, NL, 38)

### 4.3 Family and friend‐related considerations in the decision to stay

#### 4.3.1 Living close to family and friends

When their family lived close by, most interviewees mentioned staying close to family as a motive for staying or as a deterrent to migration. For example, Sandrine (BE, 33) considered living close to her family an important motive for staying, and Sara (BE, 28) decided not to move any further away from her family:

You see, another very important factor is also that my parents live very close to Maastricht. When I consider what they do for [me, my partner, and our children], that is irreplaceable. (Sandrine, BE, 33)

I have decided for the present not to live any further from my parents. Thus, not to look for jobs elsewhere […] because I want to be close to them for now. […] Yes, the reason for staying in Maastricht is directly related to the fact that my family lives close. (Sara, BE, 28)

Living close to friends has also been found to deter migration (Belot & Ermisch, 2009). The transcripts indeed showed that living close to friends was important. However, rather than being a sufficient motive on its own, living close to friends was often mentioned in combination with family‐related motives and considerations. For example, Levi (LIM, 26) specified for whom he would stay in Maastricht: ‘Colleagues … I wouldn't necessarily stay here for them … but, I would stay for my parents, family and friends’ (Levi, LIM, 26).

Nevertheless, it should be noted that some interviewees would not mind living further away from their family or friends. For example, Julia (LIM, 28) was staying in Maastricht because her partner wanted to be close to his family, and Anouk (LIM, 28) would not let her family's geographical location deter her from moving in the future:

I could easily live at a distance [from family]. […] I think it is fine to live at a distance. Yet, my partner places a lot of importance on living close to his family […] and being able to visit them weekly. (Julia, LIM, 28)

I greatly value the relationship with my direct family members […], but that would not be a decisive factor for which I would stay if I am offered opportunities elsewhere. Because, well … in the weekends you can easily visit home. (Anouk, LIM, 28)

#### 4.3.2 Family and friends in combination with other motives

Besides being mentioned on their own, family‐ and friend‐related motives would commonly be raised in combination with other factors. For example, Steffie (LIM, 33) mentioned several factors that combined social network‐related motives with motives pertaining to the physical environment: ‘I stay here because of my network and the social contacts as well as the living environment and liveability of the area’. Steffie's quote does not indicate that she valued one motive over another in her current staying behaviour, which was true in most cases where interviewees mentioned multiple motives. However, later in the interview, Steffie (LIM, 33) stated that future residential decisions may be motivated more by social network considerations than the physical environment, especially in future life stages, such as when having children:

[In the future], I think that the social network will be of more importance than the location itself. Let me take the example of having children … Imagine, I would live somewhere else, but still live close to my parents and in‐laws. Then, that would contribute to [me building a future there]. (Steffie, LIM, 33)

Furthermore, for some interviewees, a specific combination of multiple factors constituted a motive for staying. In Dutch, the interviewees would refer to this as their ‘basis’, which is difficult to translate but is closely related to a what is known in the literature as social capital and location‐specific capital acting as local ties (e.g., P. A. Fischer & Malmberg, 2001; Mulder & Malmberg, 2014). For example, Levi (LIM, 26) explained that ‘having everything in Maastricht’ constitutes the main motivation for him and his partner staying in Maastricht:

Let me put it like this … [My partner and I] both grew up in Maastricht. Admittedly, in other neighbourhoods, but we both had, and have, everything in Maastricht: family, friends, sports and work. (Levi, LIM, 26)

### 4.4 The roles of family and friends in the staying process

#### 4.4.1 Linked lives, advisors and influencers

Beyond the motives for the decision to stay, the transcripts revealed a number of other roles that family and friends played in the staying processes of the interviewees. Firstly, the concept of ‘linked lives’ (Elder, 1994; Elder et al., 2003) provided clarity in understanding how family and friends had been involved in the decision‐making processes regarding the housing trajectory. The interviewees would consider the possible effects that their personal staying decisions could have on the wellbeing of their family and friends who would be on the receiving end of the decision‐making process. These effects were particularly emphasised when parents or in‐laws required care or when the interviewees had experienced parental divorce or loss in the family. For instance, Anne (NL, 24) had moved to Maastricht for her studies and had found it very difficult living far away from her parents, sibling and grandparents ‘because it is very difficult to have to experience certain [family occasions] at a distance. […] It is not easy, not being able to visit quickly’. Furthermore, despite staying in Maastricht when leaving the parental home, Thomas (LIM, 27) had found it difficult to leave his mother:

My father […] passed away when I was [young]. […] I think my mother found us leaving the parental home very difficult. That may have caused me to […] stay with her a little longer. I felt deeply for her […] as I was the last child to leave. (Thomas, LIM, 27)

On the input end of the decision‐making process, family and friends were found to act as influencers and advisors. For example, Tom's (LIM, 29) friends had recently asked him ‘whether he will be moving to [the North] someday’, and people in Nina's (LIM, 37) social network had repeatedly asked, ‘why on earth have you stayed in Maastricht?’ In such instances, friends had, light‐heartedly, triggered the interviewees to consider moving away from Maastricht. Moreover, Steffie (LIM, 33) acknowledged that she willingly accepts advice from her partner and family members and that this may significantly influence her decisions:

On reflection, I think that my partner and family have had a great influence on my choices. […] That has always been the case for me. […] You could see [such influences] as ballast or as added value, and I have always experienced it as the latter. (Steffie, LIM, 33)

#### 4.4.2 Following in the footsteps of family and friends—or not

Secondly, the transcripts revealed that the staying and moving behaviour of family and friends had influenced the staying process of the interviewees. In some instances, the behaviours of family and friends were seen as examples or the norm for residential behaviour. This was particularly apparent in the period directly after graduation, when all interviewees had experienced their friends and peers collectively moving away from Maastricht in search of job opportunities in the Randstad. This had created a sense of ‘everyone moves, so I should too’.

For some interviewees, family members had played a role in setting preferences for moving behaviour at an earlier age. For example, Rick (NL, 40) had moved from the north of the country to Maastricht for his studies, and it seemed the natural thing to do at that age because his parents had also moved across the country for their studies:

[My parents], too, have experienced how beneficial [moving] can be for you and your personal development. For those reasons, they have always stimulated [my siblings and me] […] to spread our wings. (Rick, NL, 40)

When he was younger, Rick had not understood why people would stay in one place for a long period of time. He remembered experiencing a ‘suffocating’ feeling when he thought about lifelong staying behaviour. Nevertheless, by the time of the interview, he had been a long‐term stayer of 20 years and had started to see the beauty in staying in one place: ‘[A lifelong stayer] always has all of their friends and everything around them! Their entire lives!’ (Rick, NL, 40).

#### 4.4.3 Family and friends as social capital in times of need

Finally, family and friends are important because they constitute a large part of people's social capital. In part, the role of family and friends may be to help out in times of need. For example, Hugo's (LIM, 27), Nina's (LIM, 37) and Anne's (NL, 24) parents had offered temporary housing in the parental home during times when their adult children were searching for adequate housing. In addition, Anouk (LIM, 28), Tom (LIM, 29) and Julia (LIM, 29) expressed that they would use their social network when trying to find a job in the future, something that may be of significant value in a peripheral labour market where jobs are scarce. Indeed, Hugo (LIM, 27) and Nina (LIM, 37) had previously found jobs through their parents' networks or family businesses.

It should be noted that the collective out‐migration of friends and peers was seen by the interviewees as major losses in their local social networks. Most interviewees reflected on the period after graduation as a time in which they had to ‘start over again’ and had to invest in creating a new social network:

In that respect, you could say I have started anew. […] [After graduation], I think there were maybe two people left; other than that, everyone had left. (Anne, NL, 26)

In the beginning, [just after graduation], I had a difficult time of it. […] But after a year or two I had started to get to know the city in new ways. (Nina, LIM, 37)

The above quotes by Anne (NL, 26) and Nina (LIM, 37) reiterate that they felt, as also did Thomas (LIM, 27) and Hugo (LIM, 27), that many fewer people had stayed than had moved away and those who had moved took social capital with them. At the same time, these responses reveal the investments and efforts that the interviewees had to make to facilitate their own staying behaviour because, although they are stable, the environment is changing (e.g., Hjälm, 2014). In turn, such investments in social capital may strengthen the ties to the area and increase the likelihood of continued staying behaviour. Therefore, the findings also highlight the active roles that the interviewees played in their personal staying processes (e.g., Stockdale & Haartsen, 2018; Ye, 2018).

## 5 DISCUSSION AND CONCLUSIONS

Using semi‐structured, life‐calendar interviews, this study has explored the immobility decisions of university graduates and the roles of family and friends in their staying processes. On the individual level, the interviews have shown that a wide range of factors may trigger re‐evaluations of the decision to stay. The interviewees related key life events to the moments at which re‐evaluations of immobility decisions took place and mentioned typical demographic life events as well as unique, personal events as triggers for such re‐evaluations. These findings are in line with previous findings in the immobility literature (e.g., Haartsen & Stockdale, 2017; Hjälm, 2014; Stockdale et al., 2018; Ye, 2018) and highlight the position of nonmigration experiences in the interviewees' biographies (Barcus & Halfacree, 2018; Halfacree & Boyle, 1993).

Moreover, the interviews have revealed that some young adults frequently and consciously reflected on the decision to stay. For others, spatial immobility had been the result of a ‘lack of triggers’ or a positive feedback between settling down and continuing to stay. These findings highlight that staying processes are active (Haartsen & Stockdale, 2017; Hjälm, 2014) and affected by the length of stay (P. A. Fischer & Malmberg, 2001; Huff & Clark, 1978). Moreover, the interviewees with a history of migration may have re‐evaluated their residential situations more frequently than those who had always lived in Maastricht. This cannot be claimed with certainty using this sample, but the finding is in line with suggestions of earlier studies (Haartsen & Stockdale, 2017). Using larger samples, the literature on transnational migration has indeed discovered a link between previous and intergenerational migration experiences and the future migration behaviour of individuals (e.g., Guveli et al., 2016). Similarly, the immobility literature could extend its explorations by investigating the effects of previous and intergenerational staying experiences on future staying behaviour.

Furthermore, by focusing on the notion of ‘linked lives’, this study has reemphasised earlier findings regarding the importance of geographic proximity to family and friends in mobility decisions (e.g., Belot & Ermisch, 2009; Clark et al., 2017; Ermisch & Mulder, 2019; Michielin et al., 2008; Mulder & Malmberg, 2011, 2014; Niedomysl & Amcoff, 2011; von Reichert et al., 2014; Zorlu, 2009). For most though not all interviewees, living close to family and friends constituted a motive for staying or served as a deterrent to moving. As such, this finding highlights that immobility decisions are also motivated by other factors. Indeed, the interviews revealed a range of other motives; future research could explore the roles of intrinsic values, career trajectories and the physical and social living environment in immobility decisions in greater depth. These aspects may be investigated using the existing semi‐structured, life‐calendar interview data or with other qualitative research approaches that can reveal in‐depth information about the factors that motivate immobility decisions.

Finally, the interviews have demonstrated that family and friends play crucial roles in the staying processes of highly educated, young adults. For instance, many interviewees had considered how their residential decisions would affect the wellbeing of family members, whereas family and friends had served as advisors and influencers by providing inputs to those same decisions. Furthermore, the staying processes of young adults were ‘linked’ to those of their family and friends. Most notably, the moving and staying behaviours of family and friends had triggered re‐evaluations of the decision to stay and, at times, provided examples of preferred residential behaviour through the socialisation of attitudes. In addition, family and friends had supported and facilitated the staying process by offering accommodation and help with finding a job in times of need. Describing these roles has added some depth to the mobility literature in which the roles of family and friends have scarcely been theorised (Faist, 1997; Mulder, 2018). It should be noted here that the findings are specific to the sampled target group; the need for supportive exchange evolves over the life course and varies per individual; thus, a sample at different ages or life stages possibly reveals other sets of roles for family and friends. Based on the current findings, a particularly interesting avenue for future social network studies would be to explore how the lives of stayers are affected by the loss of social capital due to the out‐migration of others.

Overall, the life‐calendar grids proved a helpful addition to more conventional semi‐structured interviewing methods. Some interviewees expressed that the life‐calendar grid provided a convenient overview of their life course, which may have stimulated more precise recollections of the timing of life events and other occasions. Some indeed pointed out the timing of events on the life‐calendar grid during the interviews. For the author, the semi‐structured nature of the interview guide allowed more detailed information to be sought if an unforeseen topic or unexplored recollection arose. Furthermore, during the analyses, the life‐calendar grids provided a chronological visualisation of the interviewees' narratives and helped making sense of the decision‐making process by linking motives for staying with events. Thus, compared with traditional interviewing methods, the combination of life‐calendar grids and semi‐structured interview guides may have stimulated more complex, in‐depth information about the ongoing nature of the staying processes of highly educated, young stayers.

Coding the staying processes was not straightforward. In part, the difficulty in coding was caused by the lack of existing conceptualisations for the various decisions that are made at different moments in a staying process. For example, the author was expecting to code various primary and secondary motives for staying, as is common for migration motives. However, the author soon realised that the interviewees did not rank their motives according to importance but stated time‐dependent motives. This led to codes such as ‘earlier motive’, ‘later motive’ and ‘motive as a student’. Such temporal dimensions have been reported in the literature and are in line with a biographical approach to the study of nonmigration (Haartsen & Stockdale, 2017; Hjälm, 2014) but have not led to clear conceptualisations for qualitative studies on nonmigration experiences compared with, for example, the decision‐making processes of moving behaviour. Thus, a necessary condition for future immobility studies is to further unpack the staying process by means of conceptual exercises that enable the exploration of nonmigration experiences in a more comprehensive and biographical manner.

The main conclusion from this study is that immobility decisions, just like migration decisions, are diverse. A factor that greatly contributes to this diversity is the influence of ‘linked lives’. Geographic proximity has been found beneficial for strong relationships and indeed presents one way in which family and friends played a role in immobility decisions. Nevertheless, regardless of geographic proximity, family and friends were found to fulfil other crucial roles in the staying processes of highly educated, young adults. In terms of the relevance of the findings, one should recognise that the interviewees constitute strong human capital, which is a vital element for regional growth and economic development in peripheral areas such as the Dutch province of Limburg. The interviewees are examples of highly educated individuals who have stayed in a peripheral urban area despite the high out‐migration rates present among their peers. On various occasions, possessing good social capital had facilitated some interviewees to stay despite a lack of suitable jobs or housing. As such, the findings may help policymakers to understand for what reasons highly educated young adults come to stay in peripheral areas. Understanding what facilitates continued staying behaviour may contribute to policy ideas aiming to retain strong human capital.

## CONFLICT OF INTEREST

The author has no conflict of interest to declare.

## Note

^1^ In September 2003, Dutch universities implemented a bachelor‐then‐master degree structure. Interviewees who graduated before this time hold different titles, but their degrees are equivalent to what is currently considered a master's degree.
